# Examining awareness of tobacco’s oral health effects: Dentists’ role in smoking cessation among dental patients

**DOI:** 10.18332/tid/176227

**Published:** 2024-02-16

**Authors:** Betül Çalışkan Batı, Nurcan Buduneli, Pınar Meriç

**Affiliations:** 1 Department of Periodontology, Faculty of Dentistry, Ege University, İzmir, Türkiye

**Keywords:** oral health, smoking cessation, tobacco products, questionnaire

## Abstract

**INTRODUCTION:**

Tobacco products are well-known as a major risk factor for systemic and oral diseases. Dentists may play an important role in the prevention and progression of oral problems related to smoking. The aim of this study was to evaluate the level of awareness about the poor oral health effects of tobacco products and the role of dentists in smoking cessation among dental patients.

**METHODS:**

A survey containing 40 questions was prepared, and patients seeking dental treatment between June and October 2019 at the School of Dentistry, Ege University, were asked to participate. The survey included demographic variables in the first part, habits of using tobacco products in the second part, relations between smoking and oral health, and the possible role of dentists in smoking cessation in the last part. Data were tested statistically by Mann Whitney U and chi-squared tests.

**RESULTS:**

A total of 501 patients participated in the survey; more than half of the participants were non-smokers (63.7%). Cigarettes (95.06%), hookah (7.69%), e-cigarettes (2.75%), and cigars (1.65%) were the most frequently consumed tobacco products. The biggest obstacle to quitting smoking was ‘having smoker friends’. The rate of non-smokers (41.4%) agreeing that smoking is related to periodontal diseases was more than that of smokers (32.4%) (p<0.05). The most known side effect of tobacco products was halitosis (81.6%). Half of the respondents (46.7%) did not know about dentists’ role in helping them quit smoking. The rate of participants previously recommended by a dentist to quit smoking was only 36%.

**CONCLUSIONS:**

The aesthetic and social consequences of using tobacco products are well known, but smokers are substantially less aware than non-smokers of the relationship between tobacco products and oral diseases. The present findings suggest that dentists should inform their patients about the detrimental effects of tobacco products and play an active role in advising them to quit.

## INTRODUCTION

Various forms of tobacco products, including conventional cigarettes, cigars, pipe, and smokeless tobacco (chewing tobacco and snuff), continue to be the most popular hazardous substance globally. The use of tobacco products increases the risk of oral and oropharyngeal malignancies with mortality, as well as other oral health problems such as leukoplakia, halitosis, staining of the teeth and gingiva, necrotizing periodontal diseases, periodontitis, peri-implantitis, and tooth loss^1^.

Smokers are 2–7 times more likely to have periodontitis than non-smokers^2-4^. In addition, a strong association between smoking and tooth loss has been reported^5^. Furthermore, smoking cessation reduces the incidence of tooth loss by reducing the severity of periodontal disease^6-8^. Smoking is also known as the major risk factor for secondary malignancies^9^.

Dentists are probably the best-suited professionals to provide smoking cessation support to patients, as their roles and skills focus on the maintenance of oral health. Dentists are trained to treat and instruct patients on proper preventive measures to reduce oral health problems^10,11^. Frequent or regular dental appointments create an opportunity to increase the awareness of patients and encourage them to quit smoking^10,11^. Short and repetitive motivation techniques for smoking cessation are more successful than a single consultancy^12^. Frequent and regular appointments for dental interventions, particularly for quadrant-based non-surgical periodontal treatment and recall visits during the maintenance phase, can be regarded as an optimal condition for effective counselling in smoking cessation. Additionally, research shows that 60-85% of dental patients expect assistance from their dentist in quitting smoking^11-13^.

The public’s health literacy about the relationship between systemic health and tobacco products is high. Still, they have somewhat limited knowledge about the detrimental effects of tobacco products on oral health. The aesthetic and social effects of smoking, such as tooth discoloration, halitosis, and bad taste, are the most widely known and complained consequences by the patients. However, the severe effects, such as the development of periodontitis, oral cancers, and disturbed wound healing, are less known. The knowledge of smokers and non-smokers about oral problems caused by smoking was compared in previous studies. It has been reported that smokers were less aware of the relationship between periodontal diseases and wound-healing disorders than were non-smokers^13^.

It was hypothesized that patients using any type of tobacco product are less aware than non-smokers of the detrimental effects of tobacco on oral health. Therefore, the present study aimed to evaluate the knowledge about: 1) tobacco products-related risk of oral diseases; and 2) the role of dentists in smoking cessation interventions among patients seeking treatment in the School of Dentistry, Ege University, İzmir, Türkiye.

## METHODS

A survey containing 40 questions was prepared, and patients seeking treatment in the School of Dentistry, Ege University, between June and October 2019, were requested to complete the survey. The study was conducted in full accordance with ethical principles, including the World Medical Association’s Declaration of Helsinki, as revised in 2000. The study protocol was approved by the Ethics Committee of the School of Medicine, Ege University, İzmir, Türkiye (Protocol number 19-3T/37). Written informed consent was received from each patient before enrolment in the study.

Virtual forms of questionnaires were created in the Google Forms infrastructure. One tablet assigned to the study was used by a single researcher (BÇB). The questionnaire is divided into three sections: the first part of the survey included 14 questions about sociodemographic data (age, gender, body height, weight, education level, employment status, job satisfaction, breakfast habit, exercise habits, sleep patterns, alcohol consumption, systemic disease, smoking status), the second part included 11 questions about tobacco product usage status (type of tobacco product smoked; since when it has been used; number of tobacco product smoked per day; frequency and reason of smoking tobacco products; willingness, reasons, and experiences to quit; and status of taking medication or help for quitting; biggest obstacle to quit), and the last part included 15 questions about knowledge on the relationship between tobacco products and oral health problems and the possible role of dentists in smoking cessation (history of smoking among family members and close friends, smoking at home, dentists’ role in smoking cessation, relationship between oral health and tobacco product use, thoughts about dentist’s smoking, smoking cessation methods and smoking bans in public places.

### Statistical analysis

The statistical analysis was performed using SPSS software (SPSS Inc. version 21 IBM, Chicago, USA). First, the reliability of the questionnaire was tested by applying the questionnaire to a group of 20 volunteers who were not included in the study. As a result of this preliminary analysis, Cronbach’s alpha was calculated as 0.712. After the study, the Mann-Whitney U Test for continuous variables and the chi-squared test for categoric variables were used to find the possible intergroup differences or associations (p<0.05).

## RESULTS

A total of 501 dental patients aged >18 years participated in the survey. The sociodemographic characteristics of participants are summarized in Table 1. The age range of the participants was 18–82 years, and more than half of the participants were female (58.7%). More than half of the participants were nonsmokers (63.7%); employed (51.1%); not consuming alcohol (52.3%); had a university degree (56.9%); had a breakfast habit (84.2%); had no known systemic diseases (64.7%); and reported good sleep pattern (74.4%). Almost half of the participants (46.5%) did not do sports. Gender distributions of the smokers and non-smokers were similar (p>0.05). The nonsmokers were significantly older than the smokers (p=0.029). No significant difference was found between smokers’ and non-smokers’ body height and weight ratios (p>0.05).

**Table 1:** Sociodemographic characteristics of participants in a survey conducted at the School of Dentistry, Ege University, İzmir, Türkiye, June–October 2019 (N=501)

*Characteristics*	*Total (N=501) n (%)*	*Smokers (N=182) n (%)*	*Non-smokers (N=319) n (%)*	*p*
**Gender**				
Female	294 (58.7)	97 (53.3)	197 (61.8)	0.064
Male	207 (41.3)	85 (46.7)	122 (38.2)	
**Age** (years), median (IQR)	45.00 (35.00–55.00)	45.00 (32.00–52.00)	46.00 (35.00–56.00)	**0.029^†^**
**Education level**				
Not educated	4 (0.8)	0 (0)	4 (1.3)	0.234
Primary school	41 (8.2)	19 (10.4)	22 (6.9)	
Secondary school	32 (64)	9 (4.9)	23 (7.2)	
High school	139 (27.7)	54 (29.7)	85 (26.6)	
University	285 (56.9)	100 (54.9)	185 (58.0)	
**Employment status**				
Employed	256 (51.1)	113 (62.1)	143 (44.8)	<0.001*
Unemployed	245 (48.9)	69 (37.9)	176 (55.2)	
**Breakfast habit**				
Everyday	422 (84.2)	139 (76.4)	283 (88.7)	<0.001*
Often	58 (11.6)	30 (16.5)	28 (8.8)	
Rarely	28 (5.6)	12 (6.6)	16 (5.0)	
Never	6 (1.2)	1 (0.5)	5 (1.6)	
**Exercise habits**				
Everyday	50 (10.0)	8 (4.4)	42 (13.2)	0.001*
2–3 times/week	218 (43.5)	75 (41.2)	143 (44.8)	
Never	233 (46.5)	99 (54.4)	134 (42.0)	
**Sleep pattern**				
Good	374 (74.7)	116 (63.7)	258 (80.9)	<0.001*
Poor	127 (25.3)	66 (36.3)	61 (19.1)	
**Alcohol consumption**				
2–3 times/week	35 (7.0)	17 (9.3)	18 (5.6)	0.003*
Rarely	204 (40.7)	88 (48.4)	116 (36.4)	
Never	262 (52.3)	77 (42.3)	185 (58.0)	
**Systemic disease**				
Yes	177 (35.3)	46 (25.3)	131 (41.1)	<0.001*
No	324 (64.7)	136 (74.7)	188 (58.9)	
**Family history of smoking**				
Yes	301 (60.1)	140 (76.9)	161 (50.5)	<0.001*
No	200 (39.9)	42 (23.1)	158 (49.5)	
**Smokers in the family**				
Mother	52 (17.3)	25 (17.9)	27 (16.8)	<0.001*
Father	100 (33.2)	52 (37.1)	48 (29.8)	
Siblings	137 (45.5)	71 (50.7)	66 (41.0)	
Spouse	244 (81.1)	116 (82.9)	128 (79.5)	
**Smoking at home**				
Yes	157 (31.3)	95 (52.2)	62 (19.4)	<0.001*
No	344 (68.7)	87 (47.8)	257 (80.6)	
**Smoker close friends**				
Yes	384 (76.6)	167 (91.8)	217 (68.0)	<0.001*
No	117 (23.4)	15 (8.2)	102 (32.0)	

* Chi-squared test, p<0.05.

† Mann Whitney U test, p<0.05. IQR: interquartile range.

It was observed that the majority of the participants using tobacco products were working actively (62.1% vs 44.8% in non-smokers) (p<0.001). The rate of having breakfast every day was higher among non-smokers (88.7% vs 76.4% in smokers) (p<0.001). The rate of individuals doing exercise was significantly lower in the smokers (42%) compared to the non-smokers (54.4%) (p=0.001). The rate of participants with poor sleep patterns was higher among tobacco users (36.3%) than non-users (19.1%). The alcohol consumption rate was significantly higher among tobacco users (57.7% vs 42% in non-smokers) (p=0.003). The rate of participants with a known systemic disease was higher in the non-smoker group (41.1% versus 25.3% in smokers) (p<0.001). The rate of using tobacco products among family members (76.9% vs 50.5% in non-smokers) (p<0.001), indoor smoking (52.2% vs 19.4%) (p<0.001), and close friends using tobacco products (91.8% vs 68%) (p<0.001) were higher in the smoker group (Table 1).

Types and frequencies of smoking tobacco products and the responses of smokers to the questions about smoking cessation are presented in Table 2. The rates of smoking various tobacco products were as follows: cigars (1.6%), electronic cigarettes (2.7%), hookah (7.7%), and conventional cigarettes (95%). Medications for smoking cessation have been used at least once by 86.6% of smokers. A larger proportion (60%) of the respondents mentioned that the biggest obstacle to quitting smoking is ‘having smoker friends’.

**Table 2:** Types and frequencies of used tobacco products and the percentage of responses of tobacco users to the questions about smoking cessation in a survey conducted at the School of Dentistry, Ege University, İzmir, Türkiye, June–October 2019 (N=501)

*Smoking status questions*	*Responses (N=182) Percentage of preferences*
**Which one/ones do you smoke?**	
Cigarettes	95.06
Electronic cigarettes	2.75
Hookah	7.69
Cigars	1.65
	***Mean years ± SD***
**How many years have you smoked?**	16.59 ± 10.21
	***Percentage of respondents***
**How many cigarettes do you smoke in a day?**	
<10	25.5
10–20	39.5
20–30	31.7
>30	3.3
	***Mean days ± SD***
**How often do you smoke cigarettes in a week?**	6.02 ± 0.72
**How often do you smoke electronic cigarettes in a week?**	5.84 ± 0.64
**How often do you smoke hookah in a week?**	3.06 ± 3.17
	***n (%)***
**Do you consider quitting?**	
Yes	117 (64.3)
No	65 (35.7)
**Have you ever tried to quit smoking?**	
Yes	93 (51.1)
No	89 (48.9)
**Have you ever received any help to quit smoking?**	
Yes	11 (9.2)
No	109 (90.8)
**Have you ever used smoking cessation medications?**	
Yes	97 (86.6)
No	15 (13.4)
**What is the biggest obstacle for you to quit smoking?**	
Having smokers in the family	19 (10.4)
Having smoker friends	60 (33.0)
I don’t want to stop smoking	103 (56.6)

**Figure 1:**
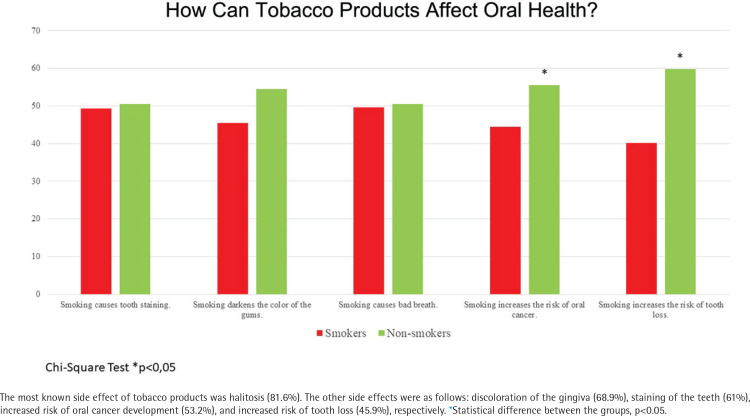
Percentage of respondents’ knowledge about the relation between tobacco products and oral diseases

**Figure 2:**
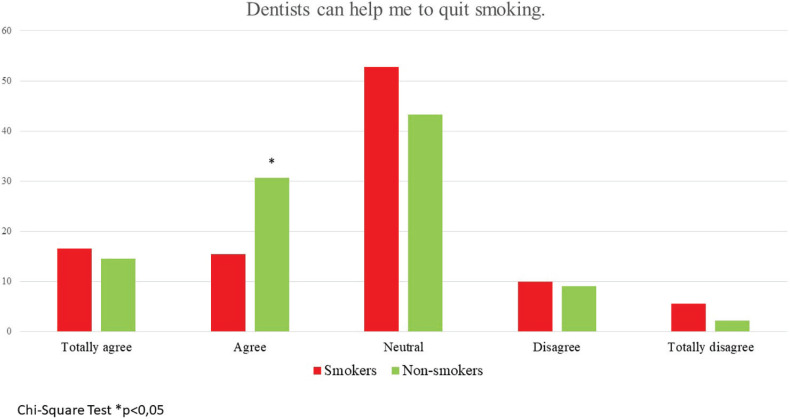
Percentage of respondents’ knowledge of the possible role of dentists in smoking cessation

Responses about the relation between tobacco products and oral health problems, dentists’ smoking habits, smoking cessation, and the possible role of dentists in smoking cessation are presented in Table 3, in Figure 1, and in Supplementary file Figures 1 and 2. Almost half of the respondents (46.7%) did not know about dentists’ role in helping them quit smoking, while 40.5% thought that dentists could help. The rate of participants previously recommended to quit smoking by a dentist was only 36% (Table 3). Most of the respondents thought that smoking is related to tooth caries (61.9%) and periodontal diseases (76.6%). The most known side effect of tobacco products was halitosis (81.6%). The other indicated side effects were as follows: discoloration of gingiva (68.9%), staining of teeth (61%), increased risk of oral cancer development (53.2%), and increased risk of tooth loss (45.9%). Non-smokers were more literate about the detrimental effects of tobacco products on oral health. Almost half of the respondents (46.7%) did not know about dentists’ possible role in helping them quit smoking, while 40.5% thought that dentists could help (Figure 2). Non-smokers (75%) chose the answer ‘The clinic smells of cigarette smoke’ approximately three times more frequently than smokers (23%). The responses of ‘His/her hands smell of cigarette smoke’ and ‘His/her clothes smell of cigarette smoke’ were approximately equally selected by both groups. At the same time, non-smokers thought that dentists should not smoke (56%), and smokers believe that this is the dentist’s personal preference (58%) (Table 3).

**Table 3:** Questions and answers about dentists’ smoking habit, smoking cessation counselling and indoor smoking in a survey conducted in the School of Dentistry, Ege University, İzmir, Türkiye, June–October 2019 (N=501)

*Questions*	*Responses*	*Smokers (N=182) n (%)*	*Non-smokers (N=319) n (%)*	*p*
**Did your dentist inform you about the detrimental effects of tobacco products and/or advise you to stop using tobacco?**	Yes	66 (36.3)	64 (20.1)	<0.001*
	No	116 (63.7)	255 (79.9)	
**Can you notice if a dentist is a smoker?**	Yes	137 (75.3)	283 (88.7)	<0.001*
	No	45 (24.7)	36 (11.3)	
**If yes, how do you notice?**	His/her hands smell of cigarette smoke	74 (40.7)	120 (37.6)	
	His/her clothes smell of cigarette smoke	78 (42.9)	124 (38.9)	0.064
	The clinic smells of cigarette smoke	30 (16.5)	75 (23.5)	
**What do you think about the dentist’s smoking habit?**	It doesn’t concern me	49 (26.9)	41 (12.8)	<0.001*
	It is his/her decision	105 (57.7)	101 (31.7)	
	He/she shouldn’t smoke	28 (15.4)	177 (55.5)	
**Do you know where you can get help to quit smoking?**	Yes	137 (77.0)	146 (67.3)	0.034*
	No	41 (23.0)	71 (32.7)	
**If yes, please specify**	‘Call 171’	94 (51.7)	62 (19.4)	
	Public hospital	34 (18.7)	66 (20.7)	0.234
	University Hospital	36 (19.8)	78 (24.5)	
	Dentist	18 (9.9)	113 (35.4)	
**What is your opinion about indoor smoking?**	It is normal	8 (4.4)	1 (0.3)	<0.001*
	You should never smoke indoors	139 (76.4)	307 (96.2)	
	You shouldn’t smoke if there is a warning sign	35 (19.2)	11 (3.4)	

* Chi-squared test, p<0.05.

## DISCUSSION

The detrimental effects of tobacco products on oral health have been investigated in numerous studies. It is important to share scientific data with the public to improve individuals’ awareness of the relationship between tobacco products and oral diseases. The present survey aimed to evaluate the level of knowledge on this issue in those patients seeking dental treatment in a university setting. The findings of the present survey indicated that smokers are less aware of the detrimental effects of tobacco products on oral health. Only one-third of smokers have been previously recommended to quit smoking by a dentist and were not expecting from dentists significant help to quit smoking. A larger proportion of the respondents mentioned that the biggest obstacle to quitting smoking is ‘having smoker friends’ and emphasized the peer effect.

According to the present findings, the rate of tobacco usage among close friends, family members, and people who smoke at home was higher among smokers. It was observed that smokers had more acquaintances who use tobacco products and were more likely to live with other smokers, and this finding was in line with those of a previous report^1^. The significant influence of peer effect on an individual’s smoking behaviors has been established before^14-18^. The social relationships of an individual play a significant role not only in starting tobacco use but also in quitting. It has been suggested that cessation programs supported by peers who had already quit smoking may increase the success rate of quitting for smokers^17^.

Patients’ knowledge regarding the relationship between smoking and dental caries was also explored in the present study. The findings in the literature on the possible relationship between smoking and dental caries are controversial. Although no proven cause-and-effect is mentioned, a meta-analysis published in 2019^19^ showed a correlation between tobacco use and increased risk of caries development. In the present study, 62% of the participants believed in a relationship between tobacco use and caries. This finding agrees with the rates from previous studies by Terrades et al.^13^, reporting 60.3%, and Sood et al.^12^, reporting 56.3%, of survey respondents who believed in a relationship between smoking and tooth caries development. On the other hand, 50.8% of the participants in the study by Al-Shammari et al.^20^ thought there was no relationship between smoking and dental caries.

Increased risk of periodontal disease development and deterioration of healing response following non-surgical and surgical periodontal treatment have been reported in smokers^21-23^. According to the present findings, 77% of the participants were aware that using tobacco products is associated with periodontal disease development. This finding is similar to the rates reported in previous studies on tobacco products and periodontal disease (64%, 80%, and 76%, respectively)^12,13,20^.

The present survey indicates that the awareness of the negative social and aesthetic consequences of smoking, such as bad breath (82%), gingival discoloration (69%), and tooth staining (61%), were relatively high. Oral malignancies are the most severe side effect of smoking in the mouth; however, only 53% of the participants were aware of this risk. Less than half of the participants (46%) knew that tobacco products increase the risk of tooth loss. This survey indicated that smokers and non-smokers differed significantly regarding awareness of the relationship between tobacco products and oral health, and this finding is in line with previous studies^12,13,20,24^. Informing patients about the relationship of tobacco products with the development of oral cancers and periodontal diseases will raise awareness among smokers. Dentists can use this fact to motivate and encourage their smoker patients to quit. Moreover, this information may help to ensure non-smokers do not start smoking.

According to the findings of the present survey, only 40% of the participants believed that dentists could assist smokers to quit. Another study reported that non-smokers and quitters were in favor of dentists’ counselling in smoking cessation (83% and 88%, respectively), but this rate was only 24% among smokers^12^. According to Ford et al.^17^, 64% of the participants believed that the dentist’s guidance could be helpful in quitting smoking. Dental clinics are excellent settings for merging smoking cessation programs as dental treatment interventions usually require regular and frequent appointments, not only during the active treatment phase but also during maintenance^25^. Smokers respond less favorably to non-surgical and surgical periodontal treatment, and there is a greater risk of recurrence in smoker patients with periodontitis^26^. Based on this evidence, it may be suggested that dentists’ enduring and caring approach, together with consistent efforts to motivate their smoker patients to quit, may significantly reduce the rate of smoking. However, the present survey indicated that only 36% of the participants had been previously advised by a dentist to quit smoking. A previous survey conducted in the UK reported that 31% of periodontologists give their patients smoking cessation advice^18^. The relatively low incidence may be explained by dentists’ lack of proper training on counselling techniques to quit smoking, feeling unqualified for this task, or being unaware that they may actively assist patients to quit. Therefore, it is highly suggested that dentists and dental hygienists should be trained in counselling techniques for smoking cessation.

Another remarkable finding of the present study is related to the smoking habits of dentists. Smoker participants seem not to be concerned about the smell of smoke in the environment of a dental clinic, whereas many non-smokers think that dentists should not smoke due to this unpleasant smell. Moreover, smoker participants stated that it is the dentist’s personal decision to smoke or not to smoke. As healthcare professionals, dentists should be aware of how they are perceived as role models in society. The dentist who informs patients about the consequences of smoking on oral health and tries to persuade patients to quit, should not be a smoker to increase persuasive power and credibility.

### Limitations

Our study has some limitations. Firstly, being in front of a healthcare professional, people may tend to respond as expected rather than reflect their actual views. Thus, there is response bias and social desirability bias. However, this fact can be considered a common limitation for most of the survey studies and can only be overcome by online surveys. Another limitation may be the digital configuration of the present survey, as people with a low level of education might have been shy about using the tablet and declined to participate in the study. Moreover, heavy smokers may not have participated in this survey due to their current smoking status.

## CONCLUSIONS

People are likely to be concerned about the unpleasant dentogingival aesthetic and social consequences of using tobacco products, and this fact creates a valuable opportunity for dental professionals to motivate their patients to quit. Moreover, there seems to be a need to emphasize the relationship between smoking and the development of periodontal diseases, oral cancer, and tooth loss. Smokers seem to be less aware of or neglect the harmful effects of tobacco products on oral health. Non-smokers’ higher awareness of the relationship between smoking and oral diseases can be regarded as an ensuring factor for them not to ever start smoking. Smokers are more tolerant of dentists’ smoking habits, whereas non-smokers are strictly against it. Remarkably, smoker dental patients expect support from their dentists to quit. Dentists’ and dental hygienists’ training on smoking cessation programs may increase the rate of quitters in society, thereby improving the oral and general health of the public.

## Data Availability

The data supporting this research are available from the corresponding author on reasonable request.
