# Supplementary material for: Examining awareness of tobacco’s oral health effects: Dentists’ role in smoking cessation among dental patients
Source: Tob Induc Dis. 2024 Feb 16;22:10.18332/tid/176227. doi: 10.18332/tid/176227 (PMC10870343; doi:10.18332/tid/176227)
Supplement: Supplementary file 1 [file TID-22-41-s1.pdf]

SUPPLEMENTARY FILE

Supplementary Figure 1 and 2 Legends:

Percentage of respondent's knowledge about relation between tobacco products and caries and periodontal disease. Most of the respondents (61.9%) thought that tobacco use is related with tooth caries, and periodontal diseases (76.6%). *Statistical difference between the groups; p<0,05.

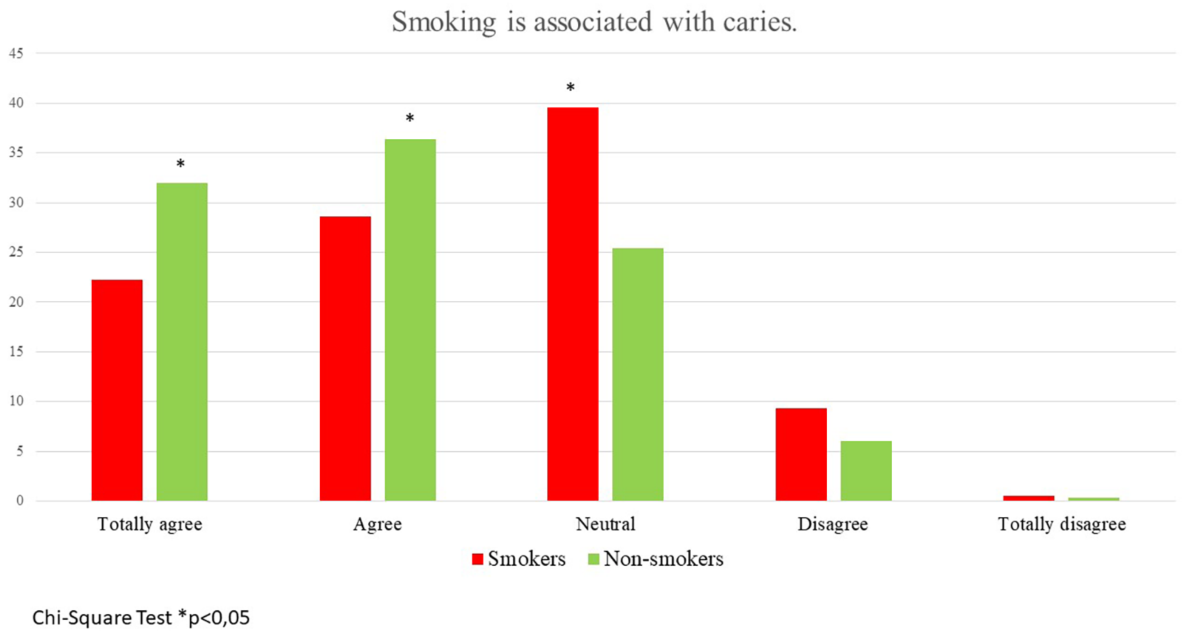

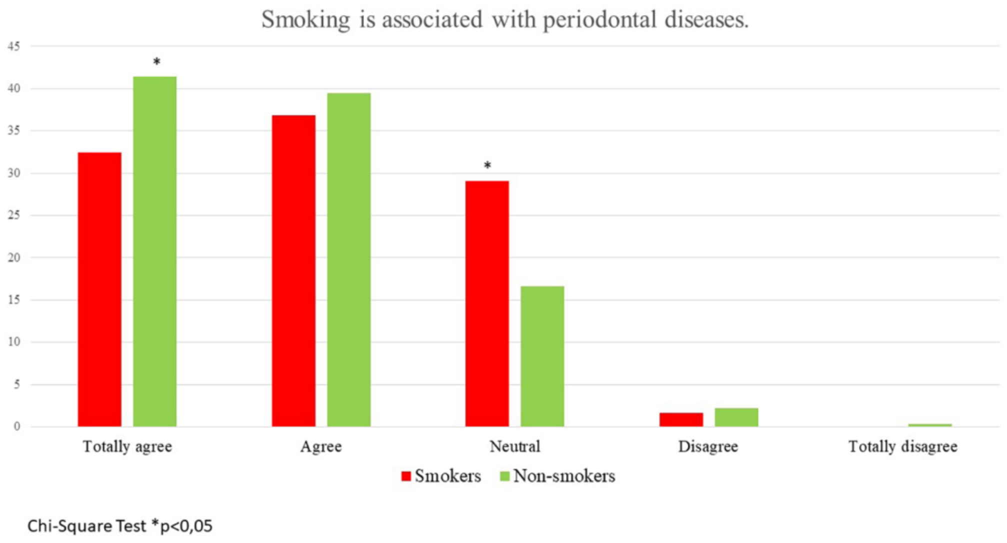

© 2024 Çalışkan Batı B. et al.
